# Exploring the Microbiota of Faba Bean: Functional Characterization of Lactic Acid Bacteria

**DOI:** 10.3389/fmicb.2017.02461

**Published:** 2017-12-12

**Authors:** Michela Verni, Changyin Wang, Marco Montemurro, Maria De Angelis, Kati Katina, Carlo G. Rizzello, Rossana Coda

**Affiliations:** ^1^Department of Soil, Plant, and Food Science, University of Bari, Bari, Italy; ^2^Department of Food and Environmental Science, University of Helsinki, Helsinki, Finland

**Keywords:** faba bean, lactic acid bacteria, starter culture, legumes, sourdough, antinutritional factors, fermentation

## Abstract

This study investigated the metabolic traits of 27 lactic acid bacteria (LAB) strains belonging to different species, previously isolated from faba bean. The activities assayed, related to technological and nutritional improvement of fermented faba bean, included peptidases, β-glucosidase, phytase, as well as exopolysaccharides synthesis and antimicrobial properties. In addition, the bacteria performance as starter cultures during faba bean fermentation on proteolysis, antioxidant potential, and degradation of condensed tannins were assessed. Fermentative profiling showed that only 7 out of 27 strains were able to metabolize D-raffinose, particularly *Leuc. mesenteroides* I01 and I57. All strains of *Pediococcus pentosaceus* exerted high PepN activity and exhibited β-glucosidase activity higher than the median value of 0.015 U, while phytase activity was largely distributed among the different strains. All the weissellas, and in lower amount leuconostocs, showed ability to produce EPS from sucrose. None of the strains did not survive the simulated gastrointestinal tract with the exception of *P. pentosaceus* I56, I76, 147, I214, having a viability of 8–9 log CFU/ml at the end of the treatment. None of the strains showed antimicrobial activity toward *Staphylococcus aureus*, while eight strains of *P. pentosaceus* exhibited a strong inhibitory activity toward *Escherichia coli* and *Listeria monocytogenes*. Generally, the doughs fermented with pediococci exhibited high amount of total free amino acids, antioxidant activity, and condensed tannins degradation. These results allowed the identification of LAB biotypes as potential starter cultures for faba bean bioprocessing, aiming at the enhancement of faba bean use in novel food applications.

## Introduction

In the last years, several studies (Randhir and Shetty, 2004; Multari et al., 2015) have been focusing on the technological and nutritional properties of faba bean (*Vicia faba* L.). Due to its nutritional quality and widespread use, this legume is considered one of the best candidates to substitute soybean and meat proteins in the diet, contributing to mitigate the environmental burden of intensive animal farming (Multari et al., 2015; Bohrer, 2017). One of the most interesting traits of faba bean is the high protein content, up to 35%, characterized by a balanced amino acids profile, especially rich in lysine. The high content of dietary fibers, vitamins, minerals, carotenoids and bioactive constituents, such as antioxidants and chemopreventive factors, further contribute to increase the nutritional quality of this legume (Randhir and Shetty, 2004).

Additionally, faba bean cultivation is beneficial to the agroecosystem in general, contributing to diversify crop rotation and enhancing the ecosystem diversity and overall sustainability (Köpke and Nemecek, 2010). Due to all these positive attributes, several efforts have been done to promote cultivation and to extend faba bean use for human and animal diet, besides its traditional consumption (Multari et al., 2015). One of the hindrances to a broader use of this legume is constituted by the presence of some so-called “ANFs,” including RFOs, protease inhibitors, phytic acid, condensed tannins, vicine, and convicine (Vilariño et al., 2009; Ray and Georges, 2010). These ANFs exert unfavorable effects on human and animal digestion, and sometimes cause pathologic conditions (Gupta, 1987). For instance, RFOs induce gastrointestinal disorders and protease inhibitors interfere with the digestion of proteins (Baucells et al., 2000). When phytate and condensed tannins content is high, they could form insoluble complexes with proteins and minerals and result in low bioavailability of minerals, proteins, and carbohydrates (Barry and McNabb, 1999). Vicine and convicine are mostly unique ANFs existing in faba bean and are toxic to individuals carrying a genetic deficiency of glucose-6-phosphate dehydrogenase (G6PD) in red blood cells, leading to the hemolytic disease called favism (Crépon et al., 2010).

Different strategies have been used to decrease the content of ANFs in faba beans and legumes, ranging from crop genetic improvement to processing (for review see Multari et al., 2015). Bioprocessing and fermentation are successful strategies to decrease the content of ANFs in faba bean while at the same time enhancing its nutritional properties, opening new application scenarios for the food industry (Coda et al., 2015, 2017b; Chandra-Hioe et al., 2016; Rizzello et al., 2016b, 2017a; Adebiyi et al., 2017). In our recent study, faba bean flour fermentation carried out through traditional backslopping method revealed the main LAB consortia and their positive influence on the nutritional quality of sourdough (Coda et al., 2017a). LAB isolated from faba bean mainly belonged to the genera *Pediococcus*, *Leuconostoc*, and *Weissella* while lower abundance of *Lactobacillus* and *Enterococcus* spp. was observed. In this study, 27 of these LAB strains previously isolated during backslopping were characterized for their technological and functional properties. At this moment, fermentation is not largely applied to legumes in food production. Moreover, in spite of the increasing novel food applications explored in the recent literature involving legumes and faba bean fermentation, there are few information on properly selected starter cultures or on their presumptive functional traits for faba bean fermentation. The design of novel food products and processes has most often involved the use of starters isolated from the matrix to be processed, potentially showing the best adaptive and competitive properties (Corbo et al., 2017), with the aim of achieving specific desired attributes (Di Cagno et al., 2013; Coda et al., 2014; Rizzello et al., 2016a). Among these, biogenic (e.g., antioxidant, antimicrobial) and probiotic activities, as well as the capacity to decrease the content of ANFs have been extensively exploited in food fermentation (Gobbetti et al., 2010).

The aim of this research was to establish the main metabolic traits and the enzymatic activities of different LAB strains, related to potential functional applications of fermented faba bean for further selection and use as starters in the food industry.

## Materials and Methods

### Microorganisms

Twenty-seven LAB strains previously isolated from Italian and Finnish faba bean sourdoughs were employed in this study (Coda et al., 2017a). All the strains, including *Enterococcus* spp. F09; *Enterococcus casseliflavus* F05; *Lactobacillus sakei* F71, F1410; *Lactococcus lactis* F55; *Leuconostoc mesenteroides* I01, I21, I57, I211; *Pediococcus* spp. I56; *Pediococcus pentosaceus* F01, F15, F77, F213, I02, I014, I76, I147, I214; *Weissella cibaria* F16, F110; and *Weissella koreensis* F111, F113, I06, I19, I148, I149 were previously identified genotypically through sequencing of the 16S rDNA gene (Coda et al., 2017a) and propagated in MRS broth (Thermo Fisher Scientific Oxoid Ltd., Basingstoke, Hampshire, United Kingdom). When characterized or used for the inoculum of doughs, the LAB strains were cultivated into MRS at 30°C for 24 h and the cells were harvested by centrifugation (10,000 × *g*, 10 min, 4°C), washed twice in 50 mmol/l sterile potassium phosphate buffer (pH 7.0), and re-suspended in tap water at the cell density of ca. 9.0 log cfu/ml. Aliquots of cell suspension were stored at -20°C before the enzymatic assays.

### Fermentative Profiling by Biolog System

The carbon-source utilization profiles of the LAB strains were determined by Biolog System (Biolog, Inc., Hayward, CA, United States) using 95 different carbon sources. Before being used for inoculating Biolog AN plates (Biolog Inc., Hayward, CA, United States), strains were grown twice on MRS broth for 24 h, and the cells harvested by centrifugation (8,000 × *g* for 10 min) and washed twice in sterile phosphate buffer 50 mmol/l pH 7.0. Then, cells were re-suspended into sterile physiological solution. Each well of the Biolog AN plates was inoculated with 100 μl bacterial suspensions adjusted to 65% transmittance as recommended by the manufacturer. Positive reactions were automatically recorded using a microplate reader with a 590-nm wavelength filter after 24 h of incubation at 30°C. Three separate experiments were carried out. Similarities between the fermentation profiles were investigated through permutation analysis, using PermutMatrixEN software (Caraux and Pinloche, 2005).

### Peptidase Activities

Peptidase activities were measured in the cell suspension (ca. 9.0 log cfu/ml) using different synthetic pNA substrates, as previously proposed by Gobbetti et al. (1999): Leucine-pNA (Leu-pNA, Sigma, as substrate for PepN, PepN EC 3.4.11.1), glutamyl-pNA (Sigma, as substrate for PepA, EC 3.4.11.7), and NCBZ-Gly-Gly-Leu-pNA (Sigma, as substrate for PepO, EC: 3.4.24.-). A reaction mixture containing 20 μl of 20 mmol/l substrate, 80 μl of 50 mmol/l Tris–HCl buffer, pH 7.5, and 100 μl of cell suspension was incubated at 30°C from 2 to 23 h, according to the time needed for the pNA release from the different substrates, preliminarily determined. The reaction was stopped by adding 500 μl of 10% acetic acid, the mixture was centrifuged at 10,000 × *g* for 10 min, and the absorbance of the supernatant, containing the released pNA, was measured at 410 nm. The analyses were performed in duplicates following the modified method of Herreros et al. (2003). One Unit (U) of peptidase activity was defined as the amount of peptidase required to produce 1 nmol of *p*-nitroaniline from substrate per 1 (PepN) or 10 (PepA and PepO) min under the assay conditions.

### β-Glucosidase Activity

β-Glucosidase activity (EC: 3.2.1.21) was measured by the release of *p*-nitrophenol from the substrate *p*-nitrophenyl-β-D-glucopyranoside (pNPG; Sigma), using a modification of the method of Di Cagno et al. (2010). The assay mixture consisted of 900 μl of 2.5 mmol/l pNPG in 0.5 M potassium phosphate buffer, pH 7.5, and 100 μl of cell suspension (ca. 9.0 log cfu/ml). After incubation at 40°C for 2 h, the reaction was stopped by heating at 95°C for 5 min. The liberated *p*-nitrophenol was determined spectrophotometrically at 410 nm. The activity of β-glucosidase was represented by the concentration of released *p*-nitrophenol, using calibration curve for *p*-nitrophenol (Sigma). The β-glucosidase activity was analyzed in triplicate.

### Phytase Activity

Phytase activity (EC: 3.1.3.8) was determined by a modified method previously proposed by De Angelis et al. (2003), based on the determination of the inorganic orthophosphate released from the phytic acid by phytases. The assay mixture contained 150 μl of cell suspensions and 600 μl of substrate, 3 mmol/l Na-phytate (Sigma) in 0.2 M Na-acetate (Merck Millipore, Finland), pH 4.0. The mixture was incubated at 45°C for 2 h and the reaction was stopped by the addition of 750 μl of 5 % trichloroacetic acid (Merck Millipore). Color reagent was prepared daily, mixing four volumes of 1.5% (w/v) ammonium molybdate (J.T. Baker, Netherlands) in 5.5% sulfuric acid (Merck Millipore) and 1 volume of 2.7 (w/v) ferrous sulfate (Merck Millipore). Seven hundred and fifty microliters of color reagent was added and the released inorganic orthophosphate was determined according to the absorbance at 700 nm by spectrophotometer. Phytase activity was determined in duplicates and 1 U of enzyme activity was defined as the amount of phytase that released 1 nmol of phosphate from sodium phytate per minute under the assay conditions (Songré-Ouattara et al., 2008).

### Exopolysaccharide (EPS) Synthesis

To investigate the synthesis of EPS, the 27 LAB strains were inoculated on MRS agar (Lab M) supplemented with 2% sucrose (Korakli et al., 2001; Katina et al., 2009). EPS synthesis was observed through the examination of slimy colonies on the plate after 48 h of incubation at 30°C. The synthesis of EPS was assessed visually and expressed with a scale -, no production; +, poor production; ++, moderate production; and +++, abundant production.

### Antimicrobial Activity

The 27 strains were tested for inhibition of potential gastrointestinal pathogens by well diffusion assay using cell culture supernatants as described by Schillinger and Lucke (1989). *S. aureus* DSM20231, *L. monocytogenes* ATCC19115, and *Escherichia coli* DSM30083, belonging to the Culture Collection of the Department of Soil, Plants and Food Science, University of Bari were used to characterize antibacterial activity. The assays were carried out using different soft agar media (5 ml) overlaid on 15 ml of agar-H_2_O (2%, wt/vol). In detail, LB (Thermo Fisher Scientific Oxoid Ltd.) was used for *E. coli* DSM30083, while M17 and BHI (Thermo Fisher Scientific Oxoid Ltd.) were, respectively, used for *S. aureus* DSM20231 and *L. monocytogenes* ATCC19115. Indicator strains were inoculated at 10^4^ CFU/ml. Wells 5 mm in diameter were cut into the agar plates and 50 μl of cell-free supernatant of the LAB cultures were placed in each well. Before the assay, the pH of the supernatants was corrected to pH 7.0 with NaOH 2 N. Plates were stored at 4°C for 4 h to allow radial diffusion of the antimicrobial substance, incubated at 37°C for 24 h and subsequently examined for zones of inhibition. Fifty microliters of sterile water and chloramphenicol (final concentration 0.1 g/l) were used as negative and positive control, respectively. All the experiments were carried out in triplicate.

### Fermentation of Faba Bean Flour: Proteolysis, Antioxidant Activity, and Degradation of Phytic Acid, Raffinose, and Condensed Tannins

The proximal composition of the faba bean flour used in this study was: moisture, 9.45 ± 0.07%; protein, 24.11 ± 0.19% of dry matter (d.m.); fat, 1.43 ± 0.01% of d.m.; total carbohydrates, 58.51 ± 0.68% of d.m. (starch, 44.83 ± 0.16% of d.m.; dietary fibers, 9.90 ± 0.36% of d.m.); and ash, 3.52 ± 0.05% of d.m. Each bacteria strain was used to ferment faba bean (*Vicia faba minor*, harvest year 2014) doughs (flour and tap water at a ratio 60:40). Before and after incubation, microbiological analysis was carried out: LAB were counted on MRS agar (Thermo Fisher Scientific Oxoid Ltd., Basingstoke, Hampshire, United Kingdom), supplemented with 0.01% of cycloheximide (Sigma Chemical Co., United States) at 30°C for 48 h, under anaerobiosis; yeasts were cultivated on Malt Agar (Oxoid) supplemented with 0.01% chloramphenicol at 25°C for 48 h, and *Enterobacteriaceae* were cultivated on VRBGA (Oxoid) at 37°C for 48 h.

At the end of the fermentation, carried out at 30°C for 24 h, the pH value of fermented doughs was determined by a pH meter (Model 507; Crison, Milan, Italy) with a food penetration probe. Two not inoculated faba bean doughs were produced in the same conditions as above described and used as controls, before (Ct0) and after incubation at 30°C for 24 h (Ctinc) WSE of fermented doughs were prepared according to Weiss et al. (1993) and used to determine proteolysis products, peptides, and amino acids. Peptide profiles were investigated by reversed-phase fast protein liquid chromatography (RF-FPLC), using a Resource RPC column and ÄKTA FPLC equipment, with a UV detector operating at 214 nm (GE Healthcare Bio-Sciences AB, Uppsala, Sweden). A sample loop of 100 μl was used. The peptides total peak area was determined with the software UNICORN 4.0 (GE Healthcare Life Sciences). A calibration curve was obtained using triptone (Oxoid) in the range 0.1–10 mg/ml. Free amino acids were analyzed by a Biochrom 30 series Amino Acid Analyzer as described by Rizzello et al. (2010).

The analysis of antioxidant activity was carried out on the methanolic extracts (ME) of the fermented faba bean flour doughs. Five grams of each sample were mixed with 50 ml of 80% methanol to get ME. The mixture was purged with nitrogen stream for 30 min, under stirring condition, and centrifuged at 4,600 × *g* for 20 min. ME were transferred into test tubes, purged with nitrogen stream, and stored at ca. 4°C before analysis. In particular, the 2,2-diphenyl-1-picrylhydrazyl (DPPH) radical scavenging activity was determined as previously described by Rizzello et al. (2010). When the antioxidant activity was determined on the WSE, the scavenging activity on DPPH-free radical was measured according to the method of Shimada et al. (1992) with some modifications (Rizzello et al., 2010). The scavenging activity of both ME and WSE was expressed as follows: DPPH scavenging activity (%) = [(blank absorbance – sample absorbance)/blank absorbance] × 100. The value of absorbance was compared with 75 ppm butylated hydroxytoluene (BHT), which was used as the antioxidant reference.

Phytic acid and raffinose concentrations were measured using Megazyme kit K-PHYT 05/07 and Raffinose/D-Galactose Assay Kit K-RAFGA (Megazyme International Ireland Limited, Bray, Ireland), respectively, following the manufacturer’s instructions.

The analysis of condensed tannin content was determined on fermented faba bean flour doughs prepared as described in Coda et al. (2015) through the vanillin assay of Price et al. (1978), using catechin as equivalents to standardize the reaction. The calibration curve was made using catechin (Sigma) and the content of condensed tannins was presented as catechin equivalents (cat.).

### Statistical Analysis

All data of biochemical analyses were obtained at least in duplicates and each replicate was analyzed twice. Data were subject to one-way ANOVA, using the IBM SPSS Statistics 26 (IBM Corporation, New York City, NY, United States) software. Data resulting from the faba bean LAB characterization were analyzed through permutation analysis using PermutMatrixEN software (Caraux and Pinloche, 2005).

## Results

### Fermentative Profiling

**FIGURE 1:**
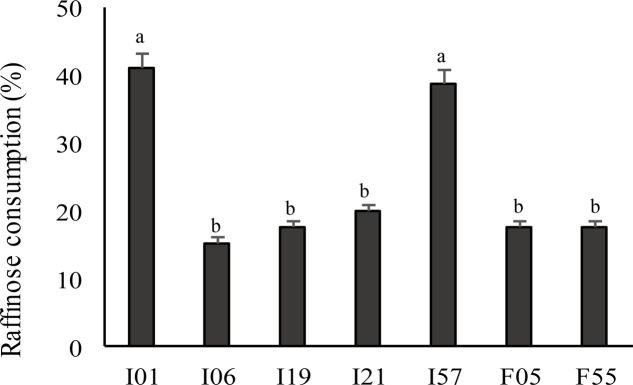
Raffinose consumption by the faba bean LAB as determined by the Biolog system. LAB were previously cultivated on MRS broth (MRS) until the stationary phase of growth (30°C for 24 h) was reached. Data are the means of three independent analyses. Error bars indicating the standard deviation are represented. ^a-d^Values with different superscript letters differ significantly (*P* < 0.05).

The profile of fermentation was determined using 95 carbon sources according to Biolog system. Overall, all the strains were able to metabolize α-D-glucose, D-fructose, D-cellobiose, mannose, maltose, maltotriose, dextrin, *N*-acetyl-D-glucosamine, D- and L-lactic acid, and its methyl esters. In particular, *P. pentosaceus* I02, I147, I014, I76, I56; *Leuc. mesenteroides* I01; and *W. koreensis* I19 showed the most intense carbohydrates consumption. *P. pentosaceus* F15 metabolized the lowest number of carbon sources, while *W*. *koreensis* I19 the highest, followed by *P. pentosaceus* I02. Only 7 out of 27 strains metabolized D-raffinose and *Leuc. mesenteroides* I01 and I57 showed the highest raffinose use (**Figure 1**).

### Peptidase Activities

The activity of PepN, PepA, and PepO was observed after 2, 6, and 23 h, respectively, at 30°C, using Leu-pNA, Glu-pNA, and NCBZ-Gly-Gly-Leu-pNA as chromogenic substrates. Among these, PepN activity was found at the highest values. As showed in **Table 1**, PepN activity ranged from 0 to 2.472 U. In particular, *P. pentosaceus* F77 showed the highest activity, with a value of 2.472 ± 0.067 U. All strains of *P. pentosaceus* exerted high PepN activity and exceeded the median value of 0.987 U, with the exception of *P. pentosaceus* I147 (0.549 U).

**Table 1:** Peptidase activities and exopolysaccharide (EPS) synthesis of the faba bean lactic acid bacteria.

Strain		Enzymatic activity			EPS
		PepN (U^A^)	PepA (U^B^)	PepO (U^B^)	Synthesis^C^
	median	0.765 ± 0.013	0.050 ± 0.004	0.010 ± 0.000	
*P. pentosaceus*	I02	2.273 ± 0.016	0.190 ± 0.020	0.020 ± 0.009	-
	I147	0.549 ± 0.007	0.250 ± 0.012	0.010 ± 0.000	-
	I014	1.977 ± 0.009	0.100 ± 0.060	0.000 ± 0.000	-
	I214	2.208 ± 0.019	0.090 ± 0.011	0.000 ± 0.000	-
	I76	1.904 ± 0.002	0.090 ± 0.021	0.000 ± 0.000	-
	F15	2.005 ± 0.074	0.230 ± 0.015	0.040 ± 0.013	-
	F77	2.472 ± 0.067	0.040 ± 0.018	0.030 ± 0.010	-
	F01	1.804 ± 0.032	0.250 ± 0.030	0.010 ± 0.000	-
	F213	1.693 ± 0.008	0.110 ± 0.038	0.010 ± 0.000	-
*Pediococcus* spp.	I56	2.033 ± 0.045	0.030 ± 0.022	0.000 ± 0.000	-
*W. koreensis*	I06	0.000 ± 0.008	0.000 ± 0.000	0.020 ± 0.001	+++
	I19	0.015 ± 0.001	0.000 ± 0.000	0.010 ± 0.000	++
	I148	0.005 ± 0.002	0.010 ± 0.008	0.020 ± 0.008	++
	I149	1.059 ± 0.044	0.190 ± 0.016	0.020 ± 0.009	+++
	F111	0.012 ± 0.000	0.010 ± 0.001	0.020 ± 0.007	+++
	F113	0.006 ± 0.001	0.000 ± 0.000	0.020 ± 0.000	+
*W. cibaria*	F110	0.000 ± 0.010	0.000 ± 0.000	0.060 ± 0.014	+++
	F16	0.011 ± 0.002	0.050 ± 0.004	0.030 ± 0.000	+
*Leuc. mesenteroides*	I01	0.781 ± 0.006	0.050 ± 0.004	0.000 ± 0.000	+
	I21	0.571 ± 0.006	0.070 ± 0.008	0.010 ± 0.000	+
	I57	0.166 ± 0.006	0.001 ± 0.001	0.000 ± 0.001	+
	I211	0.765 ± 0.013	0.050 ± 0.004	0.010 ± 0.000	+
*Lb. sakei*	F1410	1.143 ± 0.000	0.000 ± 0.000	0.010 ± 0.000	-
	F71	1.103 ± 0.013	0.020 ± 0.001	0.090 ± 0.006	-
*Enterococcus* spp.	F09	0.004 ± 0.001	1.650 ± 0.120	0.010 ± 0.000	-
*E. casseliflavus*	F05	0.000 ± 0.003	0.200 ± 0.020	0.030 ± 0.002	-
*Lc. Lactis*	F55	0.118 ± 0.006	0.160 ± 0.010	0.020 ± 0.003	-

*PepN, general aminopeptidase; PepA, glutamyl aminopeptidase; PepO, endopeptidase. ^A^One Unit (U) of peptidase activity was defined as the amount of peptidase required to produce 1 nmol of *p*-nitroaniline per minute under the assay conditions. ^B^One Unit of peptidase activity was defined as the amount of peptidase required to produce 1 nmol of *p*-nitroaniline per 10 min under the assay conditions. ^C^The EPS synthesis capacity was assessed visually and expressed with a scale –, no production; +, poor production; ++, moderate production; +++, abundant production*.

On the contrary, PepA and PepO activities were found at a lower level for all the isolates, compared to PepN activity. After 6 h incubation, 20 strains from different species showed PepA activity varying from 0 to 0.250 ± 0.030 U and only *P. pentosaceus* F09 had an activity of 1.650 ± 0.120 U (**Table 1**). The values of PepO activity ranged from 0 to 0.090 ± 0.006 U after overnight incubation (**Table 1**).

### β-Glucosidase and Phytase Activity

**FIGURE 2:**
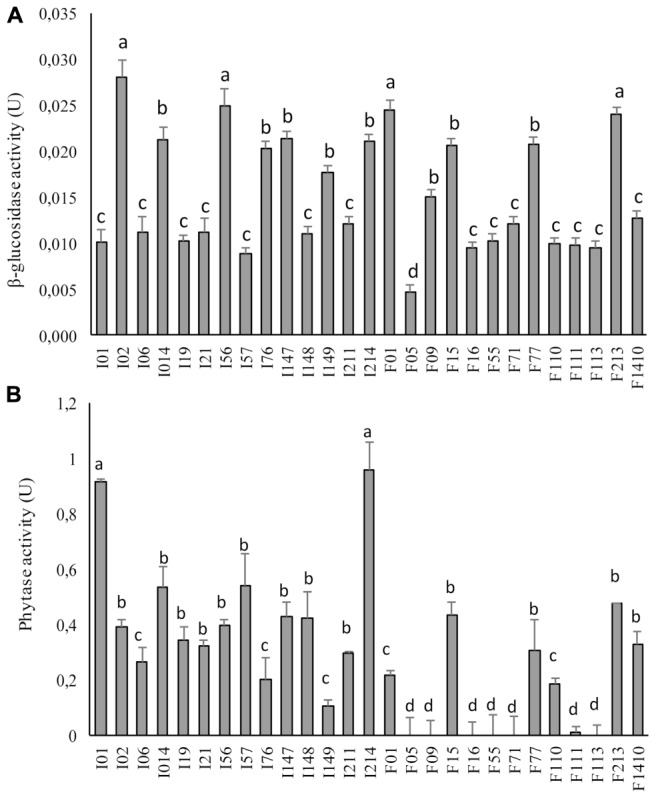
β-Glucosidase **(A)** and phytase **(B)** activities of faba bean LAB. One unit (U) of β-glucosidase activity corresponds to the release of 1 μmol/ml *p*-nitrophenol, per minute. One U of phytase activity corresponds to the release of 1 nmol of phosphate from Na-phytate per minute the under assay conditions. Data are the means of three independent analyses. Error bars indicating the standard deviation are represented. ^a-d^Values with different superscript letters differ significantly (*P* < 0.05).

The β-glucosidase activity ranged between 0.005 ± 0.001 and 0.040 ± 0.002 U (**Figure 2A**). All the strains belonging to the genus *Pediococcus* (*P. pentosaceus* I02, I147, I014, I214, I76, F15, F77, F01, F213 and *Pediococcus* spp. I56) and *W. koreensis* I149 exhibited β-glucosidase activity higher than the median value (0.015 U). In particular, *P. pentosaceus* I02, I56, F01, and F213 showed the highest activity (0.028 ± 0.002, 0.025 ± 0.002, 0.025 ± 0.001, and 0.024 ± 0.001 U, respectively).

Despite the differences, phytase activity was largely distributed among the different strains (**Figure 2B**) and ranged from 0 to 0.958 ± 0.013 U (median value of 0.288 U). In particular, *Leuc. mesenteroides* I01 and *P. pentosaceus* I214 showed the highest activity (0.912 ± 0.013 and 0.958 ± 0.150 U, respectively). Only six strains (*Lc. lactis* F55, *Enterococcus* spp. F09, *Lb. sakei* F71, *W. koreensis* F113, *W. cibaria* F16, and *E. casseliflavus* F05) were found to have no activity or activity lower than 0.108 ± 0.017 U toward Na-phytate.

### EPS Synthesis

Under the study conditions, 12 out of LAB strains showed the ability to produce EPS (**Table 1**). Specifically, all the strains belonging to the *W. koreensis* and *W. cibaria* species had a notable ability to produce EPS after 48 h incubation at 30°C. Strains of *Leuc. mesenteroides* (I01, I57, I211, I21) were also able to produce EPS, but in lower amount. No production was observed for the strains belonging to *Pediococcus* spp., *Lb. sakei*, *Enterococcus* spp., and *Lc. lactis*.

### Antimicrobial Activity

The ability of the LAB strains to inhibit potential gastrointestinal pathogens was investigated by agar-well diffusion assays, using the supernatant of the 24 h-cell cultures. Overall, *L. monocytogenes* ATCC19115 was inhibited by almost all the strains tested, with the exception of *W. koreensis* I148, I149, *Leuc. mesenteroides* I211, *Enterococcus* spp. F09, and *Lb. sakei* F71 (**Table 2**). The strongest inhibitory activity was observed for *P. pentosaceus* I014, I56, I147, I214, F77, F213, *L. lactis* F55, *W. cibaria* F110, and *W. koreensis* F11 and F113.

**Table 2:** Antimicrobial activity of the faba bean LAB (cell culture supernatants) toward potential gastrointestinal pathogens.

		*L. monocytogenes*	*S. aureus*	*E. coli*
		ATCC19115	DSM20231	DSM30083
*P. pentosaceus*	I02	+	-	++
	I147	++	-	++
	I014	++	-	++
	I214	++	-	+
	I76	+	-	++
	F15	±	-	+
	F77	++	-	+
	F01	±	-	++
	F213	++	-	+
*Pediococcus* spp.	I56	++	-	±
*W. koreensis*	I06	±	-	+
	I19	+	-	++
	I148	-	-	+
	I149	-	-	+
	F111	++	-	±
	F113	++	-	++
*W. cibaria*	F110	++	-	±
	F16	±	-	+
*Leuc. mesenteroides*	I01	±	-	±
	I21	+	-	+
	I57	±	-	±
	I211	-	-	+
*Lb. sakei*	F1410	+	-	±
	F71	-	-	±
*Enterococcus* spp.	F09	-	-	+
*E. casslifavus*	F05	±	-	++
*Lc. Lactis*	F55	++	-	+

*Inhibitory activity was scored as follows: -, no inhibition; ±, inhibition zone 1–2 mm; +, inhibition zone 3–4 mm; ++, inhibition zone diameter 5–6 mm. Data are the means of three independent analyses*.

No antimicrobial activity was found toward *S. aureus* DSM20231, while eight strains (*P. pentosaceus* I02, I014, I76, I147, F01; *W. koreensis* I19, F113; and *E. casseliflavus*) exhibited a strong inhibitory activity toward *E. coli* DSM30083 (**Table 2**).

### Faba Bean Flour Fermentation: Proteolysis, Antioxidant Activity, and Degradation of Phytic Acid, Raffinose, and Condensed Tannins

All the 27 strains were singly used to ferment faba bean doughs. Before the fermentation the pH value of the dough was 6.66, and Ct0 was characterized for cell densities of LAB, yeasts, and *Enterobacteriaceae* of 2.8 ± 0.3, 2.1 ± 0.2, and 2.0 ± 0.2 log cfu/g, respectively. After 24 h of fermentation, pH decreased in the range 4.91–5.78. In Ctinc, LAB and *Enterobacteriaceae* increased to 5.2 ± 0.1 and 5.8 ± 0.3 log cfu/g, respectively, while no increase was observed for yeasts. The lowest pH values were obtained when the fermentation was carried on with *P. pentosaceus* I76, I214, F77; *W. cibaria* F110; *Lb. sakei* F1410; and *Lc. lactis* F55; while the highest with *W. koreensis* I06, I19, I148; *W. cibaria* F16; *Enterococcus* spp. F09; *P. pentosaceus* F15; and *Lb. sakei* F71.

**FIGURE 3:**
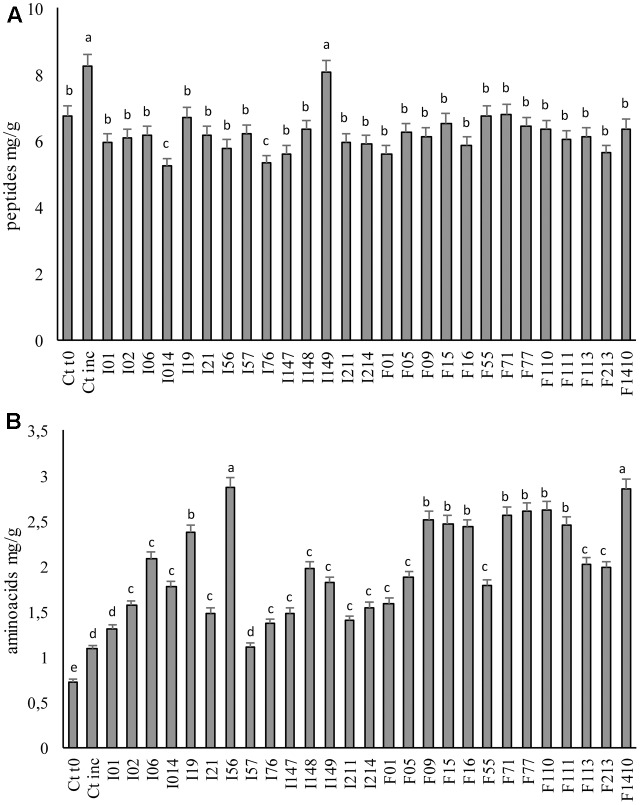
Concentration of peptides **(A)** and total free amino acids **(B)** of the fermented faba bean doughs. Two not inoculated doughs, before (Ct0) and after incubation for 24 h at 30°C (Ctinc) were the controls. Data are the means of three independent analyses. Error bars indicating the standard deviation are represented. ^a-c^Values with different superscript letters differ significantly (*P* < 0.05).

The proteolysis ability of the LAB strains was investigated through the determination of the peptides and TFAA concentrations at the end of fermentation (**Figure 3**). A significant (*P <* 0.05) increase in peptide concentration was found in Ctinc compared to Ct0, probably as the consequences of the activity of the endogenous proteolytic enzymes. When the LAB was inoculated, peptides concentration (**Figure 3A**) significantly (*P* < 0.05) decreased (with the only exception of *W. koreensis* I149). The lowest values of peptides, ranging 5.24–5.62 mg/g, were found when fermentation was carried out with *P. pentosaceus* I014, I76, F01, I147, and F213, while doughs fermented with *Lb. sakei* F71; *Lc. lactis* F55; and *W. koreensis* I19 showed the highest values (6.71–8.07 mg/g).

Before fermentation, in the Ct0, 0.73 mg TFAA/g were found (**Figure 3B**). A slight increase was observed after incubation of the not inoculated (spontaneously fermented) control dough (Ctinc), nevertheless the LAB fermentation caused the highest increases (**Figure 3B**). In particular, the doughs fermented with *Pediococcus* spp. I56 and *Lb. sakei* F1410 showed the highest TFAA concentration (2.87 mg/g). In all the other cases, an increase of two–three times the initial TFAA concentration was observed, reaching values varying from 1.11 to 2.62 mg/g.

**FIGURE 4:**
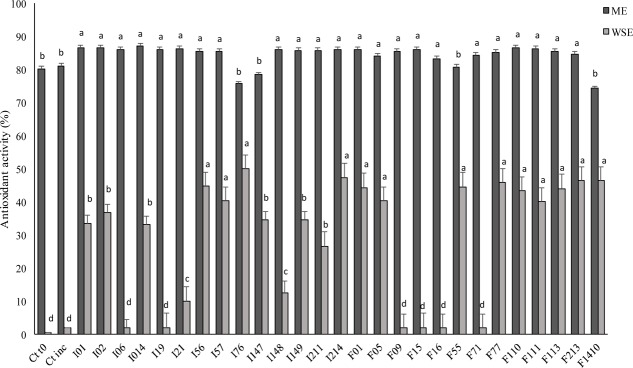
Antioxidant activity expressed as DPPH radical scavenging activity (%) determined on methanolic (ME) and water/salt-soluble (WSE) extracts from faba bean doughs inoculated with the LAB strains and fermented for 24 h at 30°C. Two not inoculated doughs, before (Ct0) and after incubation for 24 h at 30°C (Ctinc), were the controls. Data are the means of three independent analyses. Error bars indicating the standard deviation are represented. ^a-d^Values with different superscript letters differ significantly (*P* < 0.05).

The DPPH radical scavenging activity of the ME of the fermented doughs was evaluated. As shown in **Figure 4**, there were no significant (*P* > 0.05) differences among the strains, except for *P. pentosaceus* I76, I147 and *Lb. sakei* F1410 corresponding to the lowest values, 54.6, 78.4, and 74.2%, respectively. Compared to ME, the WSE (**Figure 4**) had a markedly lower radical scavenging activity, varying from 26.6 to 50.0%. Nevertheless, the increases compared to the control (0.98% of antioxidant activity) were higher than those found for the ME. The highest antioxidant activity for WSE was found when *P. pentosaceus* I56, I76, I214, and F01 were used as starters.

**FIGURE 5:**
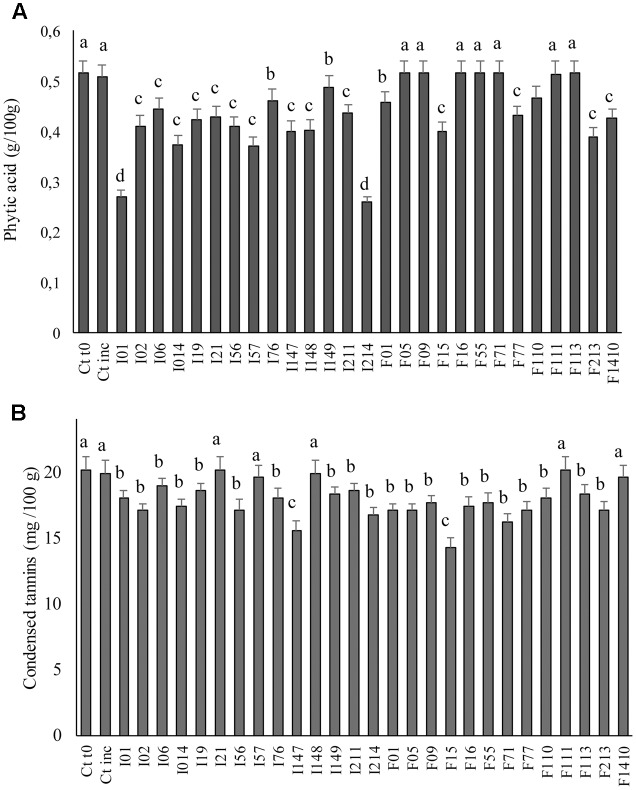
Phytic acid **(A)** and condensed tannins **(B)** concentrations in faba bean doughs inoculated with the LAB strains and fermented for 24 h at 30°C. Two not inoculated doughs, before (Ct0) and after incubation for 24 h at 30°C (Ctinc), were the controls. Data are the means of three independent analyses. Error bars indicating the standard deviation are represented. ^a-d^Values with different superscript letters differ significantly (*P* < 0.05).

A concentration of 0.51 g/100 g of phytic acid was detected in Ct0 (**Figure 5A**). After incubation without LAB inoculum no significant (*P* > 0.05) differences were found. The same was observed when *E. casseliflavus* F05, *Enterococcus* spp. F09, *W. cibaria* F16, *Lc. lactis* F55, *Lb. sakei* F71, *W. koreensis* F111, and F113 were used as starters for fermentation. Significant (*P* < 0.05) decreases were found in all the other cases. *Leuc. mesenteroides* I01 and *P. pentosaceus* I214 caused the more intense phytic acid degradation (final concentrations were 0.27 and 0.26 g/100 g, respectively).

Compared to Ct0, no significant (*P* < 0.05) differences were found in raffinose concentration of Ctinc (0.85 g/kg). A marked consumption of raffinose was found for dough started with *Leuc. mesenteroides* I01 and I57 (final concentration of 0.50 and 0.52 g/kg, respectively), while *P. pentosaceus* I21; *Lc. lactis* F55; *E. casseliflavus* F05; and *W. koreensis* I19 caused only slight (*P* < 0.05) decreases (final concentration ranging from 0.68 to 0.72 g/kg).

Condensed tannins concentration was determined on faba bean doughs after fermentation (**Figure 5B**). Compared to unfermented control (20.15 mg cat./100 g of dough), no decrease was observed for doughs fermented with *Leuc. mesenteroides* I57, I21; *W. koreensis* I148, F111; and *Lb. sakei* F1410, while the concentration of condensed tannins decreased up to ca. 30% in doughs fermented with the other strains. *P. pentosaceus* F15 was the most effective strain, followed by *P. pentosaceus* I147; *Lb. sakei* F71; and *P. pentosaceus* I214 (decrease of 23, 20, and 16%, respectively).

### Permutation Analysis

**FIGURE 6:**
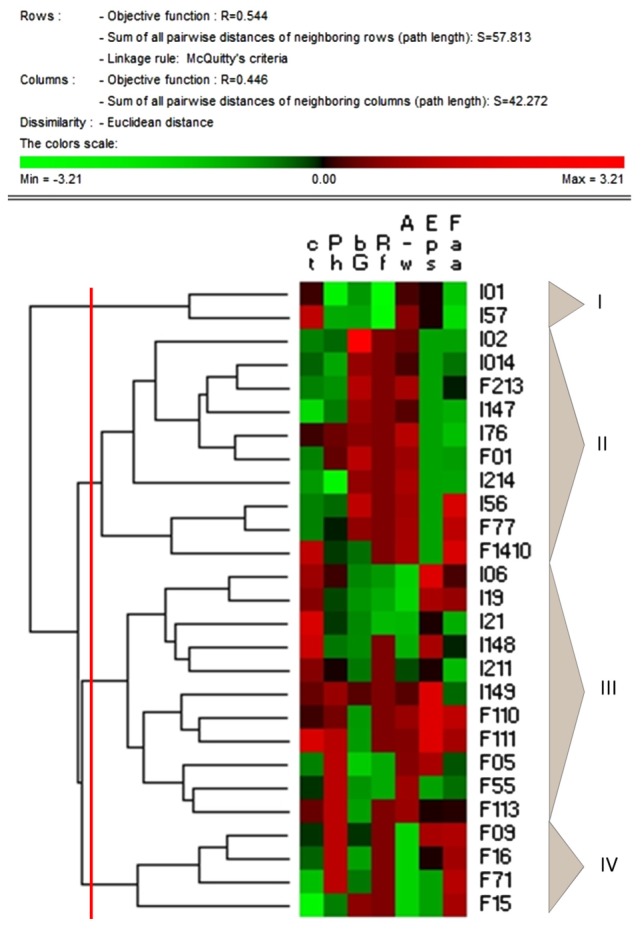
Permutation analysis of condensed tannins (cT) and phytic acid (Ph) concentrations; β-glucosidase activity (bG); raffinose concentration (Rf); antioxidant activity, as determined on the water/salt soluble extracts (A–w); EPS synthesis (Eps); free amino acids concentration (Faa). Faba bean LAB strains are indicated by codes, the name of the species is reported in the Section “Materials and Methods.” Euclidean distance and McQuitty’s criterion (weighted pair group method with averages) were used for clustering. Colors correspond to normalized mean data levels from low (green) to high (red).

Data collected from the characterization of faba bean LAB and related fermented doughs were subjected to permutation analysis (**Figure 6**). The strains were grouped into four clusters. The cluster I included the two *L. mesenteroides* strains characterized by the highest raffinose consumption (I01 and I57). The cluster II grouped eight of the nine *P. pentosaceus* strains investigated, and other two strains belonging to other species. This cluster included the major part of the strains causing the highest increases in antioxidant activity and the most intense degradation of phytic acid. Among these, *P. pentosaceus* I147, whose fermentation caused a marked decrease in condensed tannins concentration, and *P. pentosaceus* I56, also characterized by high β-glucosidase, by the ability to increase the antioxidant activity and to decrease condensed tannins and phytic acid during fermentation of faba bean dough.

The cluster III grouped EPS-producing strains belonging to *Weissella* genus, with moderate or low phytase and β-glucosidase activities, and poor ability to degrade condensed tannins and raffinose.

Cluster IV grouped strains characterized by moderate proteolytic activity (release of TFAA). Among these, *P. pentosaceus* F15 also showed high phytase and β-glucosidase activities, and an intense degradation of condensed tannins.

## Discussion

The growing importance of legumes as component of the human diet (Randhir and Shetty, 2004; Multari et al., 2015; Bohrer, 2017), and the many confirmed advantages of fermentation (Coda et al., 2015; Curiel et al., 2015; Rizzello et al., 2017a) point toward the necessity to have proper starter for bioprocessing.

In our previous studies, fermentation with LAB successfully enhanced the nutritional properties of faba bean flour and cereal-based foods fortified with such processed ingredient (Coda et al., 2015, 2017a,b; Rizzello et al., 2016b, 2017a). Based on a recent study (Coda et al., 2017a), this research aims to the characterization of the LAB strains found to be dominant in spontaneously fermented faba bean flour. Isolation was performed in faba bean doughs subjected to backslopping procedure, limiting the investigation to the biotypes showing the best competitive and adaptive features (Coda et al., 2017a). Twenty seven strains chosen on the basis of a molecular typing (Coda et al., 2017a) were characterized for their metabolic traits related to the functional and nutritional improvement of faba bean matrix. Compared to starters tailored for wheat flour fermentation, the selection of strains within the microbiota of the non-wheat matrix is a pre-requisite for rapid adaptation, and could have a positive influence on the nutritional, functional, and technological properties (Corbo et al., 2017). A phenotypic characterization of the strains was carried out through the study of the fermentative profiles. Generally, such investigation is used to understand the phenotypic manifestation of microbial environmental adaptation and as a tool in bioprocess optimization (Mozzi et al., 2016). As expected, all the faba bean LAB strains metabolize maltose, glucose, fructose, while few strains are able to use lactose and galactose. Different strains showed the ability to metabolize raffinose. According to their fermentative profiles, the concentration of raffinose in doughs fermented with *Leuc. mesenteroides* I01 and I57 markedly decreased, reaching final concentrations lower than 40% compared to controls. This α-galactoside is not degraded in the upper gastrointestinal tract due to the lack of α-galactosidase (α-Gal) activity (Teixeira et al., 2012), and, despite favoring the metabolism of beneficial intestinal microorganisms (e.g., bifidobacteria) at low concentrations, it can be fermented in the large intestine by the intestinal microbiota, causing gastrointestinal symptoms (e.g., abdominal discomfort, flatulence, and diarrhea) (Teixeira et al., 2012). Faba bean and many other legumes contain relevant concentrations of α-galactosides such as raffinose, verbascose, and stachyose which can act as ANF (Teixeira et al., 2012). Fermentation of legumes was already reported as an efficient tool for raffinose elimination in different legumes (Zamora and Fields, 1979; Connes et al., 2004). The possibility to degrade α-galactosides during fermentation can be an important selection criterion for faba bean starters (Connes et al., 2004).

Together with the lactic acid fermentation, proteolysis is considered a LAB key-feature in food biotechnology, since the degradation of native proteins is of great importance for improving the digestibility of polypeptides and bioavailability of free amino acids, but also for the release of potential bioactive peptides (with antimicrobial, antioxidant, anti-hypertensive, antitumoral activities) (Rizzello et al., 2016c). In addition, proteolysis is responsible for the organoleptic changes on taste and flavor perception, related to the release of free amino acids and their derivatives (Leroy and De Vuyst, 2004). The proteolytic system of LAB isolated from dairy and cereal environments has been largely investigated (Gobbetti et al., 2005), nevertheless, little is known about the proteolytic system of LAB isolated from other food environments, and on the effect of fermentation on legume proteins. Unlike cereal seeds, the major storage proteins in legumes, faba bean included, are globulins (mainly legumins and vicilins), characterized by abundance of leucine and glutamic acid residues (Muehlbauer and Kaiser, 2012). First, this work revealed a large distribution of the broad specificity aminopeptidase PepN, especially in *P. pentosaceus* strains, while, PepA and PepO activities were detected at a low level, although more investigation could help in addressing the role and effect of such enzymes in legume-based products.

Faba beans are rich in vicine and convicine, which are the precursors of the aglycones divicine and isouramil. These toxic derivatives are responsible, in susceptible individuals, for the hemolytic disease favism, but also for the lower nutritive value of faba beans as feed for monogastric animals. Together with the application of some processing methods (e.g., soaking, roasting, and cooking), able to contribute to the decrease of vicine and convicine concentration, the hydrolysis of such molecules and their derivatives can occur during fermentation, as a consequence of the activity of the β-glucosidases of the starter microorganisms (Cardador-Martinez et al., 2012; Rizzello et al., 2016b). In order to avoid the generation of toxic derivatives, full destruction of pyrimidine glycosides vicine and convicine before digestion is essential. According to previous findings (Zotta et al., 2007; Nuraida, 2015), high β-glucosidase activity was observed in several *P. pentosaceus* strains and in *W. koreensis*, although at lower level compared to *Lb. plantarum* DPPMAB24W, previously selected for this activity (Coda et al., 2015; Rizzello et al., 2016b). LAB β-glucosidases also play a beneficial role in the metabolism of compounds from the (iso)flavonoid family, for instance isoflavone aglycones, equol, and anthocyanins improving the functional properties of different ingredients (Di Cagno et al., 2010; Michlmayr and Kneifel, 2014).

Phytic acid is widely present in legume flours (Campos-Vega et al., 2010). Phytase, catalyzing the hydrolysis of phytic acid to myo-inositol and phosphoric acid, makes available phosphate and leads to non-metal chelator compound (Martinez et al., 1996). Phytates also reduce the digestibility of protein, starch, and lipids (Frias et al., 2003). High endogenous phytase activity was found in cereals, whereas lower activity was described for legumes (Frias et al., 2003; Steiner et al., 2007; Luo et al., 2010). Overall, lactic acidification activates cereal flour endogenous phytases due to more suitable values of pH, but, unlike cereals, it was found that the acidification conditions did not improve the activity of the legume endogenous phytases (Gustafsson and Sandberg, 1995; Coda et al., 2015). For this reason, the contribution of microbial or exogenous phytases to the degradation of phytic acid in faba bean might be relevant. LAB possess phytase activity to a certain extent, largely investigated in previous studies (De Angelis et al., 2003; Zotta et al., 2007; Songré-Ouattara et al., 2008). Among the LAB characterized in this work, *Leuc. mesenteroides* I01 and *P. pentosaceus* I214 showed the highest phytate degrading activity leading to a decrease up to 50% of the phytic acid, compared to the controls. Both the species were already efficiently used for the phytate degradation in fermented foods (Lopez et al., 2000; Oh and In, 2009; Raghavendra et al., 2011).

Recently, the interest in EPS enrichment of vegetable matrices, including legumes has been increasing (Hickisch et al., 2016; Xu et al., 2017a,b), since *in situ* synthesis during fermentation improves technological and physiochemical properties of the raw materials and derived food products (as reviewed by Galle and Arendt, 2014). In accordance with the literature data (Galle and Arendt, 2014), the highest EPS production from sucrose among the faba bean LAB was observed for different *Weissella* spp. strains.

Some LAB strains, mainly belonging to *P. pentosaceus*, showed the *in vitro* inhibition of the human pathogenic strains *E. coli* DSM30083 and *L. monocytogenes* ATCC19115. The antimicrobial activity of *P. pentosaceus* was largely documented (Porto et al., 2017).

To assess acidification, proteolysis, antioxidant activity and raffinose, phytic acid, and tannins degradation as a consequence of starter–matrix interaction, LAB strains were singly inoculated in dough, and fermented for 24 h at 30°C. The growth of faba bean LAB caused a decrease of the pH (up to 1.75 U).

Compared to controls, almost all fermented doughs were characterized by a lower total concentration of peptides. An inversely proportional correspondence with the peptidase activities was not always found since the contribution of other bacterial peptidases and endogenous proteolytic enzymes can be hypothesized. However, the increase of TFAA, as the consequence of the further peptides hydrolysis, was observed in all the fermented doughs. Compared to cereals (Lattanzi et al., 2013), the final TFAA in faba bean doughs resulted markedly higher. According to the results, a positive correlation between the proteolysis products and peptidases activities was found when *Leuc. mesenteroides* I01; *P. pentosaceus* I014, I76, I214, F01; and F213 were used as starters.

Recently, the interest for the presence of antioxidant compounds in foods has increased, according to the recognized role in the prevention mechanisms of the oxidative stresses associated with numerous degenerative aging diseases (e.g., cancer and atherosclerosis) (Adebiyi et al., 2009). The application of antioxidants in food industry is also related to the ability to delay food discoloration and deterioration, occurring as consequence of oxidative processes (Shahidi and Wanasundara, 1992). The antioxidant activity found in the ME from faba bean control dough was markedly higher than that found for common cereal doughs, probably thanks to the high total phenols content (Ragaee et al., 2006; El-Mergawi and Taie, 2014). Overall, an increase of the antioxidant activity was commonly found in fermented vegetable matrices, nevertheless, in this study, only a slight increase was found in fermented faba bean doughs compared to the control (up to 7.72%). Contrary to ME, high increases of the antioxidant activity were found on WSE as a consequence of fermentation, with relevant differences among the strains. As previously reported (Coda et al., 2012; Rizzello et al., 2017b) the antioxidant activity of the WSE is related to the increase of the TFAA concentration, but especially to peptides release. The lack of correspondence between the concentration of proteolysis degradation products and activity is not surprising, because the potential activity of the peptides strongly depends from their structure and sequence, varying on the basis of the proteolytic enzymatic activity of the different LAB strains (Coda et al., 2012; Rizzello et al., 2017b).

Faba bean contains considerable amounts of condensed tannins (Jansman and Longstaff, 1993), responsible for the formation of insoluble complexes with enzymes, other proteins, metal ions, and other macromolecules (i.e., polysaccharides) (Schofield et al., 2001). As a result, condensed tannins can reduce the nutritional value of food and feed. All the traditional processing methods including dehulling, soaking, germination, and addition of chemicals were found to be ineffective, expensive, or laborious (Jansman and Longstaff, 1993), while the effects of fermentation have been explored in different cereals and legumes with promising results (Starzynska-Janiszewska et al., 2014; Coda et al., 2015; Rizzello et al., 2016a). Some LAB enzymes, such as polyphenol oxidase and decarboxylases induced by LAB seem to be responsible for the reduction of condensed tannins during fermentation (Reddy and Pierson, 1994; Coda et al., 2015). Decreases up to ca. 30% of the initial concentration were observed in fermented faba bean doughs.

The results of this study do not lead toward the selection of a unique strain sharing all the desired tested characteristics, but allow the identification of biotypes which, singly or in pool could be useful to achieve different biotechnological goals aimed at novel products.

## Author Contributions

MV carried out the fermentations, microbiological analyses, analyses of resistance to simulated gastric/intestinal fluids, and related data elaboration. CW analyzed the enzymatic activities, EPS screening, and related data elaboration. MM performed the biochemical and Biolog analyses. MD was a scientific advisor and participated in the design of the research. KK was also a scientific advisor. CR performed the data evaluation and contributed to the writing of the article, as the coordinator of University of Bari unit. RC designed the study, performed data evaluation, and wrote the article as coordinator of University of Helsinki unit.

## Conflict of Interest Statement

The authors declare that the research was conducted in the absence of any commercial or financial relationships that could be construed as a potential conflict of interest.

## Abbreviations

ANFs: antinutritional factors
EPS: exopolysaccharide
LAB: lactic acid bacteria
ME: methanol extracts
PepA: glutamyl aminopeptidase
PepN: general aminopeptidase
PepO: endopeptidase
pNA: *p*-nitroanilide
RFOs: raffinose family oligosaccharides
RSM: reconstituted skimmed milk
TFAA: total free amino acids
WSE: water/salt-soluble extracts.

## References

[B1] Adebiyi A. O. Njobeh P. B. Adebiyi J. A. Gbashi S. Phoku J. Z. Kayitesi E. (2017). *Fermented Pulse-based Food Products in Developing Nations as Functional Foods and Ingredients, Functional Food - Improve Health through Adequate Food*, ed. Chavarri Rijeka: InTech 10.5772/intechopen.69170

[B2] Adebiyi A. P. Adebiyi A. O. Yamashita J. Ogawa T. Muramoto K. (2009). Purification and characterization of antioxidative peptides derived from rice bran protein hydrolysates. *Eur. Food Res. Technol.* 228 553–563. 10.1111/j.1365-2621.2006.01379.x

[B3] Barry T. N. McNabb W. C. (1999). “The effect of condensed tannins in temperate forages on animal nutrition and productivity,” in *Tannin Livestock Human Nutrition*, ed. Brooker J. D. (Canberra, ACT: Australian Centre for International Agricultural Research), 30–35.

[B4] Baucells F. Perez J. F. Morales J. Gasa J. (2000). Effect of a a-galactosidase supplementation of cereal-soya-bean-pea diets on the productive performances, digestibility and lower gut fermentation in growing and finishing pigs. *Anim. Sci.* 71 157–164. 10.1017/S1357729800054989

[B5] Bohrer B. M. (2017). Review: nutrient density and nutritional value of meat products and non-meat foods high in protein. *Trends Food Sci. Technol.* 65 103–112. 10.1016/j.tifs.2017.04.016

[B6] Campos-Vega R. Loarca-Piña G. Oomah B. D. (2010). Minor components of pulses and their potential impact on human health. *Food Res. Int.* 43 461–482. 10.1016/j.foodres.2009.09.004

[B7] Caraux G. Pinloche S. (2005). PermutMatrix: a graphical environment to arrange gene expression profiles in optimal linear order. *Bioinformatics* 21 1280–1281. 10.1093/bioinformatics/bti141 15546938

[B8] Cardador-Martinez A. Maya-Ocaña K. Ortiz-Moreno A. Herrera-Cabrera B. E. Dávila-Ortiz G. Múzquiz M. (2012). Effect of roasting and boiling on the content of vicine, convicine and L-3, 4-dihydroxyphenylalanine in *Vicia faba* L. *J. Food Qual.* 35 419–428. 10.1111/jfq.12006

[B9] Chandra-Hioe M. V. Wong C. H. M. Arcot J. (2016). The potential use of fermented chickpea and faba bean flour as food ingredients. *Plant Foods Hum. Nutr.* 71 90–95. 10.1007/s11130-016-0532-y 26880215

[B10] Coda R. Di Cagno R. Gobbetti M. Rizzello C. G. (2014). Sourdough lactic acid bacteria: exploration of non-wheat cereal-based fermentation. *Food Microbiol.* 37 51–58. 10.1016/j.fm.2013.06.018 24230473

[B11] Coda R. Kianjam M. Pontonio E. Verni M. Di Cagno R. Katina K. (2017a). Sourdough-type propagation of faba bean flour: dynamics of microbial consortia and biochemical implications. *Int. J. Food Microbiol.* 248 10–21. 10.1016/j.ijfoodmicro.2017.02.009 28242419

[B12] Coda R. Melama L. Rizzello C. G. Curiel J. A. Sibakov J. Holopainen U. (2015). Effect of air classification and fermentation by *Lactobacillus plantarum* VTT E-133328 on faba bean (*Vicia faba* L.) flour nutritional properties. *Int. J. Food Microbiol.* 193 34–42. 10.1016/j.ijfoodmicro.2014.10.012 25462921

[B13] Coda R. Rizzello C. G. Pinto D. Gobbetti M. (2012). Selected lactic acid bacteria synthesize antioxidant peptides during sourdough fermentation of cereal flours. *Appl. Environ. Microbiol.* 78 1087–1096. 10.1128/AEM.06837-11 22156436 PMC3273010

[B14] Coda R. Varis J. Verni M. Rizzello C. G. Katina K. (2017b). Improvement of the protein quality of wheat bread through faba bean sourdough addition. *Food Sci. Technol.* 82 296–302. 10.1016/j.lwt.2017.04.062

[B15] Connes C. Silvestroni A. Leblanc J. G. Juillard V. Savoy de Giori G. Sesma F. (2004). Towards probiotic lactic acid bacteria strains to remove raffinose-type sugars present in soy-derived products. *Lait* 84 207–214. 10.1051/lait:2003030

[B16] Corbo M. R. Racioppo A. Monacis N. Speranza B. (2017). “Commercial starters or autochtonous strains? That is the question”,” in *Starter Cultures in Food Production*, eds Speranza B. Bevilacqua A. Corbo M. R. Sinigaglia M. (Hoboken, NJ: John Wiley & Sons), 174–198. 10.1002/9781118933794.ch10

[B17] Crépon K. Marget P. Peyronnet C. Carrouéea B. Aresec P. Ducb G. (2010). Nutritional value of faba bean (*Vicia faba* L.) seeds for feed and food. *Field Crops Res.* 115 329–339. 10.1016/j.fcr.2009.09.016

[B18] Curiel J. A. Coda R. Centomani I. Summo C. Gobbetti M. Rizzello C. G. (2015). Exploitation of the nutritional and functional characteristics of traditional Italian legumes: the potential of sourdough fermentation. *Int. J. Food Microbiol.* 196 51–61. 10.1016/j.ijfoodmicro.2014.11.032 25522057

[B19] De Angelis M. Gallo G. Corbo M. R. McSweneedy P. L. H. Faccia M. Giovine M. (2003). Phytase activity in sourdough lactic acid bacteria: purification and characterization of a phytase from *Lactobacillus sanfranciscensis* CB1. *Int. J. Food Microbiol.* 87 259–270. 10.1016/S0168-1605(03)00072-2 14527798

[B20] Di Cagno R. Coda R. De Angelis M. Gobbetti M. (2013). Exploitation of vegetables and fruits through lactic acid fermentation. *Food Microbiol.* 33 1–10. 10.1016/j.fm.2012.09.003 23122495

[B21] Di Cagno R. Mazzacane F. Rizzello C. G. Vincentini O. Silano M. Giuliani G. (2010). Synthesis of isoflavone aglycones and equol in soy milks fermented by food-related lactic acid bacteria and their effect on human intestinal Caco-2 cells. *J. Agric. Food Chem.* 58 10338–10346. 10.1021/jf101513r 20822177

[B22] El-Mergawi R. Taie H. A. A. (2014). Penolic composition and antioxidant activity of raw seeds, green seeds sprouts of ten faba bean (*Vicia faba*) cultivars consumed in Egypt. *Int. J. Pharm. Biol. Sci.* 5 609–617.

[B23] Frias J. Doblado R. Antezana J. R. Vidal-Valverde C. (2003). Inositol phosphate degradation by the action of phytase enzyme in legume seeds. *Food Chem.* 81 233–239. 10.1016/S0308-8146(02)00417-X 7002470

[B24] Galle S. Arendt E. K. (2014). Exopolysaccharides from sourdough lactic acid bacteria. *Crit. Rev. Food Sci. Nutr.* 54 891–901. 10.1080/10408398.2011.617474 24499068

[B25] Gobbetti M. De Angelis M. Corsetti A. Di Cagno R. (2005). Biochemistry and physiology of sourdough lactic acid bacteria. *Trends Food Sci. Technol.* 16 57–69. 10.1016/j.tifs.2004.02.013

[B26] Gobbetti M. Di Cagno R. De Angelis M. (2010). Functional microorganisms for functional food quality. *Crit. Rev. Food Sci. Nutr.* 50 716–727. 10.1080/10408398.2010.499770 20830633

[B27] Gobbetti M. Lanciotti R. De Angelis M. Corbo M. R. Massini R. Fox P. (1999). Study of the effects of temperature, pH, NaCl, and a w on the proteolytic and lipolytic activities of cheese-related lactic acid bacteria by quadratic response surface methodology. *Enzyme Microb. Technol.* 25 795–809. 10.1016/S0141-0229(99)00110-6

[B28] Gupta Y. (1987). Anti-nutritional and toxic factors in food legumes: a review. *Plant Food Hum. Nutr.* 37 201–228. 10.1007/BF01091786 2853348

[B29] Gustafsson E. L. Sandberg A. S. (1995). Phytate reduction in brown beans (*Phaseolus vulgaris* L.). *J. Food Sci.* 60 149–152. 10.1111/j.1365-2621.1995.tb05626.x

[B30] Herreros M. A. Fresno J. M. Prieto M. J. G. Tornadijo M. E. (2003). Technological characterization of lactic acid bacteria isolated from Armada cheese (a Spanish goats’ milk cheese). *Int. Dairy J.* 13 469–479. 10.1016/S0958-6946(03)00054-2

[B31] Hickisch A. Beer R. Vogel R. F. Toelstede S. (2016). Influence of lupin-based milk alternative heat treatment and exopolysaccharide-producing lactic acid bacteria on the physical characteristics of lupin-based yogurt alternatives. *Food Res. Int.* 84 180–188. 10.1016/j.foodres.2016.03.037

[B32] Jansman A. J. M. Longstaff M. (1993). “Nutritional effects of tannins and vicine/covicine in legume seeds,” in *Proceedings of the Second International Workshop on “Antinutritional Factors (ANFS) in Legume Seeds”*, eds Poel A. F. B. van der Huisman J. Saini H. S. (Wageningen: Pers Wageningen), 301–316.

[B33] Katina K. Maina N. H. Juvonen R. Flander L. Johansson L. Virkki L. (2009). *In situ* production and analysis of *Weissella confusa* dextran in wheat sourdough. *Food Microbiol.* 26 734–743. 10.1016/j.fm.2009.07.008 19747607

[B34] Köpke U. Nemecek T. (2010). Ecological services of faba bean. *Field Crops Res.* 115 217–233. 10.1016/j.fcr.2009.10.012

[B35] Korakli M. Rossmann A. Gänzle M. G. Vogel R. F. (2001). Sucrose metabolism and exopolysaccharide production in wheat and rye sourdoughs by *Lactobacillus sanfranciscensis*. *J. Agric. Food Chem.* 49 5194–5200. 10.1021/jf0102517 11714302

[B36] Lattanzi A. Minervini F. Di Cagno R. Diviccaro A. Antonielli L. Cardinali G. (2013). The lactic acid bacteria and yeast microbiota of eighteen sourdoughs used for the manufacture of traditional Italian sweet leavened baked goods. *Int. J. Food Microbiol.* 163 71–79. 10.1016/j.ijfoodmicro.2013.02.010 23558189

[B37] Leroy F. De Vuyst L. (2004). Lactic acid bacteria as functional starter cultures for the food fermentation industry. *Trends Food Sci. Technol.* 15 67–78. 10.1016/j.tifs.2003.09.004

[B38] Lopez H. W. Ouvry A. Bervas E. Guy C. Messager A. Demigne C. (2000). Strains of lactic acid bacteria isolated from sour doughs degrade phytic acid and improve calcium and magnesium solubility from whole wheat flour. *J. Agric. Food Chem.* 48 2281–2285. 10.1021/jf000061g 10888537

[B39] Luo Y. Xie W. Cui Q. (2010). Effects of phytases and dehulling treatments on in vitro iron and zinc bioavailability in faba bean (*Vicia faba* L.) flour and legume fractions. *J. Food Sci.* 75 C191–C198. 10.1111/j.1750-3841.2009.01490.x 20492225

[B40] Martinez C. Ros G. Periago M. J. Lopez G. Ortuno J. Rincon F. (1996). Phytic acid in human nutrition. *Food Sci. Technol. Int.* 2 201–209.

[B41] Michlmayr H. Kneifel W. (2014). β-Glucosidase activities of lactic acid bacteria: mechanisms, impact on fermented food and human health. *FEMS Microbiol. Lett.* 352 1–10. 10.1111/1574-6968.12348 24330034

[B42] Mozzi F. Raya R. R. Vignolo G. M. (2016). *Biotechnology of Lactic Acid Bacteria: Novel Applications*. Hoboken, NJ: John Wiley & Sons.

[B43] Muehlbauer F. J. Kaiser W. J. (2012). *Expanding the Production and Use of Cool Season Food Legumes: A Global Perspective of Peristent Constraints and of Opportunities and Strategies for Further Increasing the Productivity and Use of Pea, Lentil, Faba Bean, Chickpea and Grasspea in Different Farming System*, Vol. 19 Dordrecht: Springer Science & Business Media, 118.

[B44] Multari S. Stewart D. Russell W. R. (2015). Potential of fava bean as future protein supply to partially replace meat intake in the human diet. *Compr. Rev. Food Sci. Food Saf.* 14 511–522. 10.1111/1541-4337.12146

[B45] Nuraida L. A. (2015). Review: health promoting lactic acid bacteria in traditional Indonesian fermented foods. *Food Sci. Hum. Wellness* 4 47–55. 10.1016/j.fshw.2015.06.001

[B46] Oh N. In M. (2009). Phytate degradation by *Leuconostoc mesenteroides* KC51 cultivation in soymilk. *Afr. J. Biotechnol.* 8 3023–3026.

[B47] Porto M. C. W. Kuniyoshi T. M. Azevedo P. O. S. Vitolo M. Oliveira R. P. S. (2017). *Pediococcus* spp.: an important genus of lactic acid bacteria and pediocin producers. *Biotechnol. Adv.* 35 361–374. 10.1016/j.biotechadv.2017.03.004 28284993

[B48] Price M. L. Van Scoyoc S. Butler L. G. (1978). A critical evaluation of the vanillin reaction as an assay for tannin in sorghum grain. *J. Agric. Food Chem.* 26 1214–1218. 10.1021/jf60219a031

[B49] Ragaee S. Abdel-Aalb E. M. Noamanc M. (2006). Antioxidant activity and nutrient composition of selected cereals for food use. *Food Chem.* 98 32–38. 10.1016/j.foodchem.2005.04.039

[B50] Raghavendra P. Ushakumari S. Halami P. (2011). Phytate-degrading *Pediococcus pentosaceus* CFR R123 for application in functional foods. *Benef. Microbes* 2 57–61. 10.3920/BM2010.0031 21831790

[B51] Randhir R. Shetty K. (2004). Microwave-induced stimulation of L-DOPA, phenolics and antioxidant activity in faba bean (*Vicia faba*) for Parkinson’s diet. *Process Biochem.* 39 1775–1784. 10.1016/j.procbio.2003.08.006

[B52] Ray H. Georges F. (2010). A genomic approach to nutritional, pharmacological and genetic issues of faba bean (*Vicia faba*): prospects for genetic modifications. *GM Crops Food* 1 99–106. 10.4161/gmcr.1.2.11891 21865878

[B53] Reddy N. R. Pierson M. D. (1994). Reduction in antinutritional and toxic components in plant foods by fermentation. *Food Res. Int.* 27 281–290. 10.1016/0963-9969(94)90096-5

[B55] Rizzello C. G. Lorusso A. Montemurro M. Gobbetti M. (2016a). Use of sourdough made with quinoa (*Chenopodium quinoa*) flour and autochthonous selected lactic acid bacteria for enhancing the nutritional, textural and sensory features of white bread. *Food Microbiol.* 56 1–13. 10.1016/j.fm.2015.11.018 26919812

[B56] Rizzello C. G. Lorusso A. Russo V. Pinto D. Marzani B. Gobbetti M. (2017a). Improving the antioxidant properties of quinoa flour through fermentation with selected autochthonous lactic acid bacteria. *Int. J. Food Microbiol.* 241 252–261. 10.1016/j.ijfoodmicro.2016.10.035 27810447

[B57] Rizzello C. G. Losito I. Facchini L. Katina K. Palmisano F. Gobbetti M. (2016b). Degradation of vicine, convicine and their aglycones during fermentation of faba bean flour. *Sci. Rep.* 6:32452. 10.1038/srep32452 27578427 PMC5006014

[B58] Rizzello C. G. Nionelli L. Coda R. De Angelis M. Gobbetti M. (2010). Effect of sourdough fermentation on stabilisation, and chemical and nutritional characteristics of wheat germ. *Food Chem.* 119 1079–1089. 10.1016/j.foodchem.2009.08.016

[B59] Rizzello C. G. Tagliazucchi D. Babini E. Rutella G. S. Taneyo Saa D. L. Gianotti A. (2016c). Bioactive peptides from vegetable food matrices: research trends and novel biotechnologies for synthesis and recovery. *J. Funct. Foods* 27 549–569. 10.1016/j.jff.2016.09.023

[B60] Rizzello C. G. Verni M. Koivula H. Montemurro M. Seppa L. Kemell M. (2017b). Influence of fermented faba bean flour on the nutritional, technological and sensory quality of fortified pasta. *Food Funct.* 8 860–871. 10.1039/C6FO01808D 28128388

[B61] Schillinger U. Lucke F. K. (1989). Antibacterial activity of *Lactobacillus sakei* isolated from meat. *Appl. Environ. Microbiol.* 55 1901–1906. 2782870 10.1128/aem.55.8.1901-1906.1989 PMC202976

[B62] Schofield P. Mbugua D. M. Pell A. N. (2001). Analysis of condensed tannins: a review. *Anim. Feed Sci. Technol.* 91 21–40. 10.1016/S0377-8401(01)00228-0

[B63] Shahidi F. Wanasundara J. P. D. (1992). Phenolic antioxidants. *Crit. Rev. Food Sci. Nutr.* 32 67–103. 10.1080/10408399209527581 1290586

[B64] Shimada K. Fujikawa K. Yahara K. Nakamura T. (1992). Antioxidative properties of xanthan on the antioxidation of soybean oil in cyclodextrin emulsion. *J. Agric. Food Chem.* 40 945–948. 10.1021/jf00018a005

[B66] Songré-Ouattara L. T. Mouquet-Rivier C. Icard-Vernière C. Humblot C. Diawara B. Guyot J. P. (2008). Enzyme activities of lactic acid bacteria from a pearl millet fermented gruel (ben-saalga) of functional interest in nutrition. *Int. J. Food Microbiol.* 128 395–400. 10.1016/j.ijfoodmicro.2008.09.004 18937991

[B67] Starzynska-Janiszewska A. Stodolak B. Mickowska B. (2014). Effect of controlled lactic acid fermentation on selected bioactive and nutritional parameters of tempeh obtained from unhulled common bean (*Phaseolus vulgaris*) seeds. *J. Sci. Food Agric.* 94 359–366. 10.1002/jsfa.6385 24037686

[B68] Steiner T. Mosenthin R. Zimmermann B. Greiner R. Roth S. (2007). Distribution of phytase activity, total phosphorus and phytate phosphorus in legume seeds, cereals and cereal by-products as influenced by harvest year and cultivar. *Anim. Feed Sci. Technol.* 133 320–334. 10.1016/j.anifeedsci.2006.04.007

[B69] Teixeira J. S. McNeill V. Ganzle M. G. (2012). Levansucrase and sucrose phoshorylase contribute to raffinose, stachyose, and verbascose metabolism by lactobacilli. *Food Microbiol.* 31 278–284. 10.1016/j.fm.2012.03.003 22608234

[B70] Vilariño M. Métayer J. P. Crépon K. Duc G. (2009). Effects of varying vicine, convicine and tannin contents of faba bean seeds (*Vicia faba* L.) on nutritional values for broiler chicken. *Anim. Feed Sci. Technol.* 150 114–121. 10.1016/j.anifeedsci.2008.08.001

[B71] Weiss W. Vogelmeier C. Görg A. (1993). Electrophoretic characterization of wheat grain allergens from different cultivars involved in bakers’ asthma. *Electrophoresis* 14 805–816. 10.1002/elps.11501401126 8404825

[B72] Xu Y. Coda R. Shi Q. Tuomainen P. Katina K. Tenkanen M. (2017a). Exopolysaccharides production during the fermentation of soybean and fava bean flours by *Leuconostoc mesenteroides* DSM 20343. *J. Agric. Food Chem.* 65 2805–2815. 10.1021/acs.jafc.6b05495 28326776

[B73] Xu Y. Wang Y. Coda R. Säde E. Tuomainen P. Tenkanen M. (2017b). *In situ* synthesis of exopolysaccharides by *Leuconostoc* spp. and *Weissella* spp. and their rheological impacts in fava bean flour. *Int. J. Food Microbiol.* 248 63–71. 10.1016/j.ijfoodmicro.2017.02.012 28258980

[B74] Zamora A. F. Fields M. L. (1979). Nutritive quality of fermented cowpeas (*Vigna sinensis*) and chickpeas (*Cicer arietinum*). *J. Food Sci.* 44 234–236. 10.1111/j.1365-2621.1979.tb10049.x

[B75] Zotta T. Ricciardi A. Parente E. (2007). Enzymatic activities of lactic acid bacteria isolated from Cornetto di Matera sourdoughs. *Int. J. Food Microbiol.* 115 165–172. 10.1016/j.ijfoodmicro.2006.10.026 17174429

